# The Effect of Bony Parameters on the Pediatric Knee: Normal versus Anterior Cruciate Ligament Injury versus Tibial Spine Avulsion Fracture

**DOI:** 10.1055/s-0036-1597663

**Published:** 2016-12-22

**Authors:** Kenneth A. Shaw, Brian S. Dunoski, Neil Mardis, Donna Pacicca

**Affiliations:** 1 Department of Orthopaedic Surgery, Dwight David Eisenhower Army Medical Center, Fort Gordon, Georgia; 2 Department of Pediatric Radiology, Children's Mercy Hospital, Kansas City, Missouri; 3 Department of Pediatric Orthopaedic Surgery, Children's Mercy Hospital, Kansas City, Missouri

**Keywords:** pediatric, knee anatomy, anterior cruciate ligament, tibial spine avulsion fracture

## Abstract

**Purpose** Anterior cruciate ligament (ACL) injuries can present as a ligamentous disruption or avulsion fracture of the tibial spine in pediatric patients. Differences in knee morphometric parameters have been investigated between pediatric cohorts with ACL disruptions and tibial spine avulsion fractures. However, no study to date has compared morphometric parameters in patients with tibial spine avulsion fracture against a control population.

**Methods** A retrospective review of pediatric patients undergoing knee magnetic resonance imaging (MRI) studies was performed, identifying 15 patients with tibial spine avulsion fracture between January 1, 2009, and January 1, 2013. Inclusionary criteria consisted of patients who sustained an acute tibial spine avulsion fracture and had MRI examination. The MRI studies were analyzed by a pediatric musculoskeletal radiologist, who measured identified bony parameters, and results were compared with an age-matched control group and a skeletally immature cohort with ligamentous disruption of the ACL. Data were analyzed using unpaired *t* test and logistic regression.

**Results** Cohorts included 15 patients with a tibial spine avulsion fracture, 39 with an ACL disruption, and 28 in the age-matched control group. The tibial spine group demonstrated no significant differences in bony parameters when compared with the control group, but had significantly wider tibial eminence widths in comparison to the ACL group (2.92 cm [0.4] versus 2.71 cm [0.27]; *p* = 0.040). Additionally, this finding was predictive of tibial spine avulsion injury when assessed by logistic regression.

**Conclusions** Pediatric patients who sustain a tibial spine avulsion fracture exhibit significantly wider tibial eminences when compared with the cohort with ACL injuries. This indicates a possible biomechanical explanation for differences in ACL injury patterns that should be examined in future, prospective analyses.

Injury to the pediatric anterior cruciate ligament (ACL) has been characterized as either midsubstance ligamentous disruption or avulsion fracture of the tibial spine. 1 2 3 4 Previous studies have identified possible differences in injury patterns based upon the loading conditions of the knee and/or strength of the interface between the ACL, femur, and tibia. 5 6 Additionally, the osseous morphology of the knee, specifically the intercondylar notch width index (NWI), has been found to be statistically different between cohorts of patients, with significantly smaller NWI in midsubstance ACL disruptions when compared with tibial spine avulsion fractures. 7 The purpose of this study was to expand the investigations between osseous morphologic parameters in cohorts of skeletally immature patients with an ACL injury versus a tibial spine avulsion fracture and an age-matched control.

## Methods

Upon receiving approval from the Institutional Review Board, all radiology reports for magnetic resonance images (MRIs) obtained of the knee between January 1, 2009, and January 1, 2013, at a tertiary pediatric hospital were reviewed. MRI studies were performed using 3- and 1.5-T magnets with imaging slices 0.5 mm in width, acquired in the parasagittal plane and reconstructed in the coronal and axial planes using either turbo spine-echo or proton density SPACE sequences. Patients with an ACL injury or tibial spine avulsion fracture were identified by the International Classification of Diseases, Ninth Revision codes (844.2 and 823.80, respectively) and were cross-referenced against the MRI review. Reports that indicated the presence of an acute injury to the ACL or a tibial spine avulsion fracture were identified for epidemiologic review. Information including month of injury, age, gender, laterality of injury, and concomitant injury was collected.

### Study Group

**Fig. 1:**
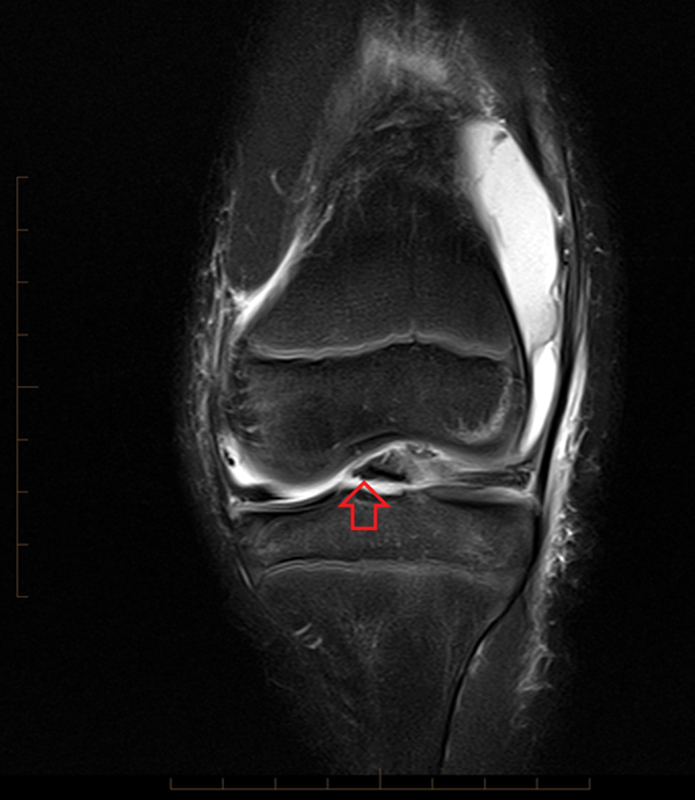
Coronal image from T2 sequence demonstrating a tibial spine avulsion fracture (arrow).

Inclusionary criteria for study participation for the tibial spine avulsion fracture group consisted of patients with acute injuries demonstrated on MRI, defined by the presence of an associated effusion. Patients were excluded from participation if they were without the identified injury, were older than 18 years, or had imaging studies performed at outside facilities. Following identification, the MRI sequences were analyzed by a pediatric musculoskeletal radiologist. Upon confirming the presence of the tibial spine avulsion fracture ( Fig. 1 ), measurements were performed of the tibia and the femur utilizing the annotation tools of the InteleViewer picture archiving and communication system (Intelerad Medical Systems, Montreal, Canada).

Measurements were performed according to be a previously published protocol. 8 Tibial parameters included the posterior slope and depth of the medial and lateral tibial plateaus, assessed from the sagittal imaging sequences. Coronal plane images were then analyzed to assess the width, height, and volume of the tibial eminences. Femoral measurements included intercondylar notch width, bicondylar width, NWI, medial and lateral femoral condyle width, and intercondylar notch volume.

### Control Groups

Following identification of the study participants, two control groups were employed for comparison. The first group consisted of data from a previously published cohort of skeletally immature patients with an isolated ACL disruption. 8 Additionally, a cohort was identified of children with a statistically similar median age ( *p* < 0.05) to the study group, in whom a knee MRI was performed as part of their routine care and who demonstrated an intact ACL and no associated ligamentous injury. MRI sequences were analyzed and measurements performed for each group utilizing the defined tibial and femoral morphometric parameters.

### Statistical Analyses

Statistical analyses were performed using SAS statistical software version 9.2 (SAS Institute Inc., Cary, North Carolina, United States). Unpaired *t* tests were performed for each measurement, comparing the tibial spine avulsion fracture group to the controls. Additionally, logistic regression was employed, using a model consisting of medial tibial plateau slope, medial tibial plateau depth, NWI, intercondylar notch volume, tibial eminence volume, and tibial eminence height and width to determine if these variables were predictive of an increased risk of sustaining an ACL injury. Statistical significance was predetermined as *p* < 0.05.

## Results

A total of 57 patients were identified as sustaining a tibial spine avulsion fracture between January 2009 and January 2013. Of these, an MRI was obtained in 25 patients, with 10 of these studies were performed at outside facilities, leaving a total of 15 patients (average age 14.3 ± 3.5 years, 5 skeletally mature, 12 boys and 3 girls). Previously identified cohorts of patients with an isolated ACL disruption ( *n* = 39, average age 14.245 ± 2.08 years, 18 girls and 21 boys) and an age-matched control group ( *n* = 28, average age 14.29 ± 1.08 years, 14 girls and 14 boys) were used for comparison.

Group statistics and results of the between-group *t* tests for the various radiographic parameters are summarized in Table 1 for all patient cohorts. In comparison with the control group, patients with a tibial spine avulsion fracture demonstrated no statistically significant differences in the assessed morphologic parameters. In comparison with the midsubstance ACL disruption group, tibial eminence width was significantly wider in the tibial spine avulsion fracture group (2.92 [0.4] versus 2.71 [0.27]; *p* = 0.040). Additionally, tibial eminence width was found to be predictive of injury on logistic regression analysis ( *p* = 0.044) with an odds ratio of 0.063.

**Table 1:** Group statistics and results of unpaired *t* test for three patient cohorts: tibial spine avulsion fracture, midsubstance ACL disruption, and control group (measured in cm)

Parameter	Group	Mean (SD)	*P* value
Notch width	Tibial spine	1.91 (0.21)	
	ACL	1.83 (0.28)	0.36
	Control	1.93 (0.26)	0.86
Notch width index	Tibial spine	0.27 (0.03)	
	ACL	0.26 (0.03)	0.35
	Control	0.28 (0.03)	0.49
Notch volume	Tibial Spine	5.99 (1.75)	
	ACL	6.36 (1.74)	0.48
	Control	6.21 (1.65)	0.67
MTP slope	Tibial spine	2.86 (2.38)	
	ACL	3.54 (2.42)	0.35
	Control	3.89 (1.57)	0.095
LTP slope	Tibial spine	3.28 (2.83)	
	ACL	3.73 (2.46)	0.56
	Control	3.25 (2.68)	0.98
MTP depth	Tibial spine	0.24 (0.14)	
	ACL	0.27 (0.09)	0.42
	Control	0.21 (0.09)	0.46
LTP depth	Tibial spine	0.01 (0.04)	
	ACL	0.02 (0.06)	0.61
	Control	0.01 (0.03)	0.98
TP width	Tibial spine	7.25 (0.75)	
	ACL	7.23 (0.64)	0.92
	Control	7.20 (0.63)	0.81
TE height	Tibial spine	0.84 (0.16)	
	ACL	0.93 (0.15)	0.06
	Control	0.93 (0.12)	0.07
TE width	Tibial spine	2.92 (0.40)	
	ACL	2.71 (0.27)	0.04 a
	Control	2.77 (0.34)	0.20
TE volume	Tibial spine	2.15 (0.84)	
	ACL	2.21 (0.71)	0.76
	Control	2.17 (0.60)	0.93
Bicondylar width	Tibial spine	7.12 (0.60)	
	ACL	7.03 (0.62)	0.62
	Control	7.00 (0.63)	0.54

Abbreviations: ACL, anterior cruciate ligament; LTP, lateral tibial plateau; MTP, medial tibial plateau; SD, standard deviation; TE, tibial eminence; TP, tibial plateau.

a Denotes statistical significance at *p* < 0.05.

## Discussion

The influence of osseous morphologic parameters on ACL injuries has been a well-studied topic in the adult literature. Previous investigations have identified an association with ACL injury and stenosis of the intercondylar notch, 9 10 11 12 13 increased anterior-posterior knee laxity, 13 14 increased posterior sloping of the tibial plateau, 15 16 17 shallow tibial plateau, 16 decreased femoral condyle width, 18 19 increased intercondylar notch volume, 20 and decreased volume of the tibial eminence. 21 As it relates to the skeletally immature knee, few studies have investigated the influence of these osseous parameters, finding correlations with stenosis of the NWI and increased slope of the medial tibial plateau with ligamentous ACL injuries. 17 22 We previously reported a correlation between stenosis of the NWI and ligamentous ACL injury, but also found that the influence of these parameters changed based on the specific injury pattern. 8

Kocher et al performed an age- and sex-matched cohort comparison with tibial spine avulsion fractures and midsubstance ACL disruptions. 7 They found a correlation between decreased NWI and midsubstance ACL disruption, despite the existence of substantial overlap in the NWI values between the groups. Due to this overlap, they could not establish a threshold NWI value for midsubstance ACL disruption. The clinical significance of NWI remains elusive; neither notch width nor NWI has been found to demonstrate a positive correlation with three-dimensional intercondylar notch volume, 20 23 a parameter that exhibits a positive correlation with respect to ACL volume. 24

Previous studies have suggested loading patterns and biomechanical properties of the skeletally immature knee as key variables in differentiating between ACL injury patterns. Noyes et al demonstrated that slower loading rates of the primate ACL complexes, consisting of the ACL and the femoral and tibial insertions, resulted in preferential failure by tibial spine avulsion. 5 Woo et al performed a cadaveric analysis of the tensile properties of ACL complex and found that the linear stiffness, ultimate load, and energy absorption of the ACL complex were inversely related with specimen age, with younger specimens demonstrating higher values. 6 Additionally, younger specimens were found to fail preferentially by bony avulsion of the tibial spine when tested in the anatomic orientation.

In the current study, we found a correlation between increased tibial eminence width and tibial spine avulsion fractures. The position of the tibial eminences with regard to the tibial insertion of the ACL has been well discussed in the literature. 25 26 27 The tibial insertion has been found to have a variable shape, ranging from oval to triangular. 28 Ferretti et al found the medial tibial eminence had a constant relationship with the central aspect of the ACL insertion, located 5.7 mm anterior to the apex of the medial eminence. 27 If the width between the medial and lateral eminences were correlated with the insertional mediolateral area of the ACL, this would, biomechanically, explain the correlation with the tibial spine avulsion fractures in the tensile properties of the ACL. Further research should assess the relationship, if any exists, between the insertional anatomy of the ACL and the width of the tibial eminences.

There are several limitations to our study. The retrospective design has inherent biases. The possibility of selection bias could be present considering only 26% of the identified patients in the tibial spine avulsion group met the inclusionary criteria. Geographical patient differences may be present, as the cohorts consisted of a homogenous group of children from the Midwestern United States, potentially explaining the difference from previous studies as well as limiting the extrapolation of the study results. Additionally, included imaging studies were restricted to studies performed at our institution to minimize measurement error from the influence of magnets of varying strength as well as different imaging protocols. Patients with established care in our hospital network may be more likely to be included in the study. The control group was also limited by the number of patients requiring MRI evaluation of their knee for reasons of nonligamentous origin. Experimental bias of the measurements also cannot be excluded.

## Conclusion

In summary, differentiating between injury patterns in the pediatric knee is a complicated venture. This study demonstrated a statistically significant difference in the width of the tibial eminences between the tibial spine avulsion and midsubstance ACL disruption cohorts with wider tibial eminences being predictive of tibial spine avulsion fractures. This information suggests a biomechanical implication of the ACL tibial footprint in the difference of injury patterns in the skeletally immature patient. Future research is needed to further characterize this relationship as well as to investigate the potential for neuromuscular training to decrease the incidence of these injuries in the skeletally immature.
